# COVID-19 strategy in organizing and planning orthopedic surgery in a major orthopedic referral center in an area of Italy severely affected by the pandemic: experience of the Department of Orthopedics, University of Padova

**DOI:** 10.1186/s13018-020-01740-4

**Published:** 2020-07-23

**Authors:** P. Ruggieri, G. Trovarelli, A. Angelini, E. Pala, A. Berizzi, D. Donato

**Affiliations:** Department of Orthopedics and Orthopedic Oncology, University of Padova, Via Giustiniani 2, 35128 Padova, Italy

**Keywords:** COVID-19, Orthopedics and COVID, Italy and COVID, Padova COVID experience

## Abstract

**Background:**

According to the required reorganization of all hospital activities, the recent COVID-19 pandemic had dramatic consequences on the orthopedic world. We think that informing the orthopedic community about the strategy that we adopted both in our hospital and in our Department of Orthopedics could be useful, particularly for those who are facing the pandemic later than Italy.

**Methods:**

Changes were done in our hospital by medical direction to reallocate resources to COVID-19 patients. In the Orthopedic Department, a decrease in the number of beds and surgical activity was stabilized. Since March 13, it has been avoided to perform elective surgery, and since March 16, non-urgent outpatient consultations were abolished. This activity reduction was associated with careful evaluation of staff and patients: extensive periodical swab testing of all healthcare staff and swab testing of all surgical patients were applied.

**Results:**

These restrictions determined an overall reduction of all our surgical activities of 30% compared to 2019. We also had a reduction in outpatient clinic activities and admissions to the orthopedic emergency unit. Extensive swab testing has proven successful: of more than 160 people tested in our building, only three COVID-19 positives were found, and of over more than 200 surgical procedures, only two positive patients were found.

**Conclusions:**

Extensive swab test of all people (even if asymptomatic) and proactive tracing and quarantining of potential COVID-19 positive patients may diminish the virus spread.

## Background

The recent COVID-19 pandemic had dramatic reflexes on the organization of the health care system and hospitals worldwide; of course, it also had consequences on the orthopedic world and it required reorganization of all the hospital activities.

We decided to report the experience in our Department of Orthopedics and Orthopedic Oncology at the University of Padova in Italy for several reasons. First of all, Italy was the first country in Europe suffering from the COVID-19 pandemic. Second, Padova is one of the major towns in Veneto, and the region Veneto in Italy has been one of the most heavily affected by the COVID-19 pandemic. Besides these, chronologically, the two first areas, where unfortunately COVID-19 began in Europe, were in our two Italian regions Lombardia and Veneto. More precisely, the first person that died of coronavirus infection was on February 21 in a small village named Vo’ Euganeo, which is very close to Padova. The other focus of the beginning infection in Italy was in Codogno, another small town close to Milan in Lombardia. Finally, ours is the major Hospital in Padova, and it was elected since the start as the referral center for all COVID-19 patients.

In our hospital, thanks to an appropriate political organization when the infection began, actions were performed to try to face the virus spread. We think that informing the orthopedic community about the strategy that we adopted both in our hospital and in our Department of Orthopedics could be useful, particularly for those who are facing the pandemic later than Italy.

## Methods

This is a report of the current experience in orthopedics during the COVID-19 pandemic at the Department of Orthopedics of the University of Padova, Veneto, Italy. Our Orthopedic Department is part of a huge hospital in Padova that includes about 1800 beds, collected in two areas: one university hospital of over 1500 beds and another smaller hospital of about 290 beds, called Sant’Antonio Hospital.

Changes were done in the Orthopedic Department as well as in other departments of the university hospital by medical direction (D.D., one of our co-authors, our Medical Director) in order to minimize resources reallocating anesthesiologists, nurses, and medical assistants to dedicate to COVID-19 patients in new ICUs, semi-intensive units, and recovery units. Since the start, it was decided that Sant’Antonio Hospital is COVID free, whereas the university hospital could accept patients who were probably positive. Of course, areas were identified into the university hospital to be fully dedicated to COVID patients: these areas and these units were increasing over the times, according to the need. Also, the strategy involved a relevant increase in the number of beds of the intensive care unit (ICU): we used to have 44 beds, while 27 beds were added, so going to a total number of 71 beds available during COVID-19 pandemic. Also, part of the Cardiac Surgery Unit was adapted to become an ICU during the COVID-19 pandemic. Another crucial decision was to divide the emergency unit into two areas: one a COVID area for all suspected cases and another COVID-free area.

Consequent to this, there was the reorganization of the Orthopedic Department (directed by P.R., the first author) that is part of the university hospital. Usually, our department had 88 beds. However, a dramatic decrease in the number of beds and reduction of the surgical activity was necessary during this COVID-19 pandemic: it was decided that the number of beds had to be 32. We usually have three operating rooms (ORs) available, if required, even 24 h daily; now, with the restrictions in the COVID-19 infection, we have two ORs working 12 h a day with one OR available 24 h for emergencies. Since March 13, it has been avoided to perform elective surgery. Consequently, despite the activity of our department before COVID-19 including all types of orthopedic surgeries, most of these required to be cut off and our current activity includes only trauma surgery (such as fractures) and musculoskeletal tumor surgery (such as malignant bone tumors, soft tissue sarcomas, metastases, and pathologic or impending fractures) (Table 1).

**Table 1:** Orthopedics surgery activities performed before and during COVID-19, when was recommended to perform only urgent or emergent cases

	Before COVID-19	During COVID-19
Trauma surgery	Yes	Yes
Fractures	Yes	Yes
Muscle-tendon injuries and sport traumas	Yes	No
Elective surgeries	Yes	No
Total hip arthroplasties	Yes	No
Total knee arthroplasties	Yes	No
Total shoulder arthroplasties	Yes	No
Shoulder recon and arthroscopies	Yes	No
Hip and knee arthroscopies	Yes	No
All other elective procedures	Yes	No
Musculoskeletal tumors	Yes	Yes
Malignant	Yes	Yes
Metastases	Yes	Pathologic and impending fractures
Benign	Yes	Selected cases (e.g., pathologic fractures)

There was also a reduction in outpatient clinic activity: since March 16, our medical direction abolished non-urgent outpatient consultations, allowing only acute evaluation (e.g., oncologic check and post-op check) to reduce the virus spread.

This activity reduction was associated with a careful evaluation of staff and patients. On one side, extensive swab testing with periodically retesting of all healthcare staff (physicians, nurses, sanitary assistants, etc.) was applied. On the other side, every day, all patients attending consultation are scanned with a thermometer and have to wear surgical masks during their permanence in the hospital. Moreover, extensive swab testing of all surgical patients was also performed. Summarizing, our procedure for admission in our department of surgical patients during COVID-19 was based on five categories:

1. Patients admitted through the Emergency Department: Always nasopharyngeal swab in urgent modality. A room in the ward is reserved for patients waiting for the result of the nasopharyngeal swab.
2. Patients requiring surgical treatment in an emergency: Nasopharyngeal swab is done in urgent modality, but the patient has surgery before having the results. Patients are treated as potential COVID-19 positive: all staff have to use FFP2 instead of a surgical mask, and OR is sanitized after surgery.
3. Patients transferred from other hospitals: Need to have nasopharyngeal swab done with the result before our admission
4. Pediatric patients: Same as point 1, 2, and 3 but also their parent/parents “caregiver” need to have nasopharyngeal swab.
5. Patients coming from their home for planned surgeries in priority class (e.g., tumors): They need to have had nasopharyngeal swab before admission or if not possible at admission (see 1).

## Results

**Fig. 1:**
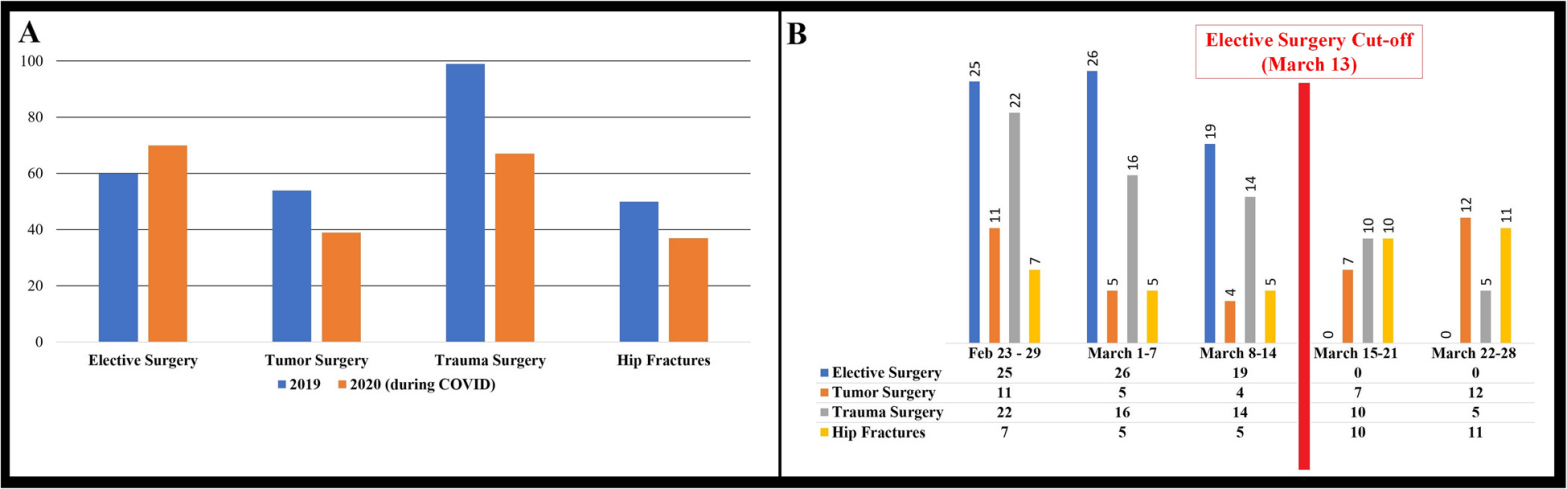
Reduction of surgical activity. There was a reduction in monthly surgeries performed comparing 2019 and 2020. **b** Surgical activity performed weekly in 2020 divided by types of surgery, considering the day of elective surgery cutoff on March 13th

These restrictions determined an overall reduction of all our surgical activities of 30% compared to 2019 (Table 2). We initially also had a relative reduction in the number of hip fractures. However, there were no actual differences in terms of low-energy fractures since they occur in elderly patients following an accidental fall at home. In contrast, high-energy fractures dramatically decrease by 85%. Tumor surgery, once we excluded basically benign tumors, was decreased by over 28% (Fig. 1a, b). Since March 6, it has been recommended to reduce elective surgery: we performed elective surgery until March 13 when restrictions were applied (Fig. 1a, b). Moreover, a decrease in surgical activity was also due to delay in intervention depending on the need to obtain swab test results before surgery and elongation of operating timing for patients treated in an emergency before having the results.

**Table 2:** How our practice has changed comparing the same period in 2019 and 2020 (from February 24 to March 31)

	2019	2020
Elective surgery	90	60 until 13 March
Trauma surgery	148	106
Hip fractures	49	38 (22% less)
Low-energy fractures	59	59
High-energy fractures	35	5 (85% less)
Children fractures	5	4
Tumor surgery and biopsies	54	39 (28% less)
Total	292	205 (30% less)

**Fig. 2:**
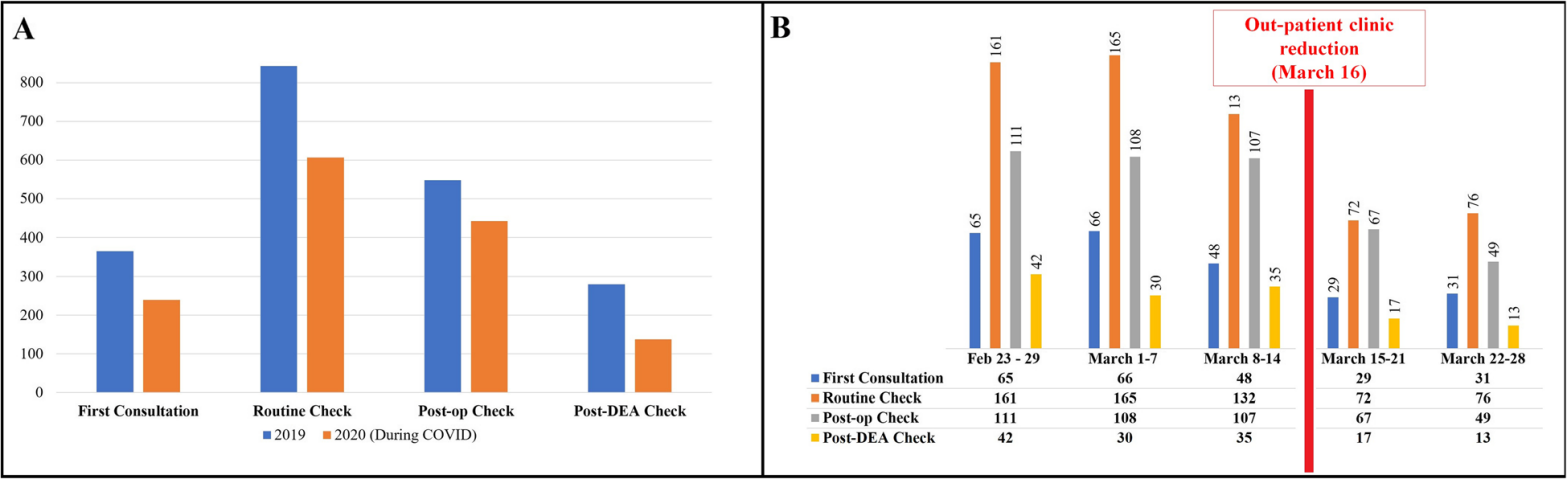
Reduction of outpatient clinic. **a** There was a reduction in monthly outpatient clinic performed comparing 2019 and 2020. **b** Different types of consultations performed weekly in the outpatient clinic: after March 16th only urgent consultations were admitted

**Fig. 3:**
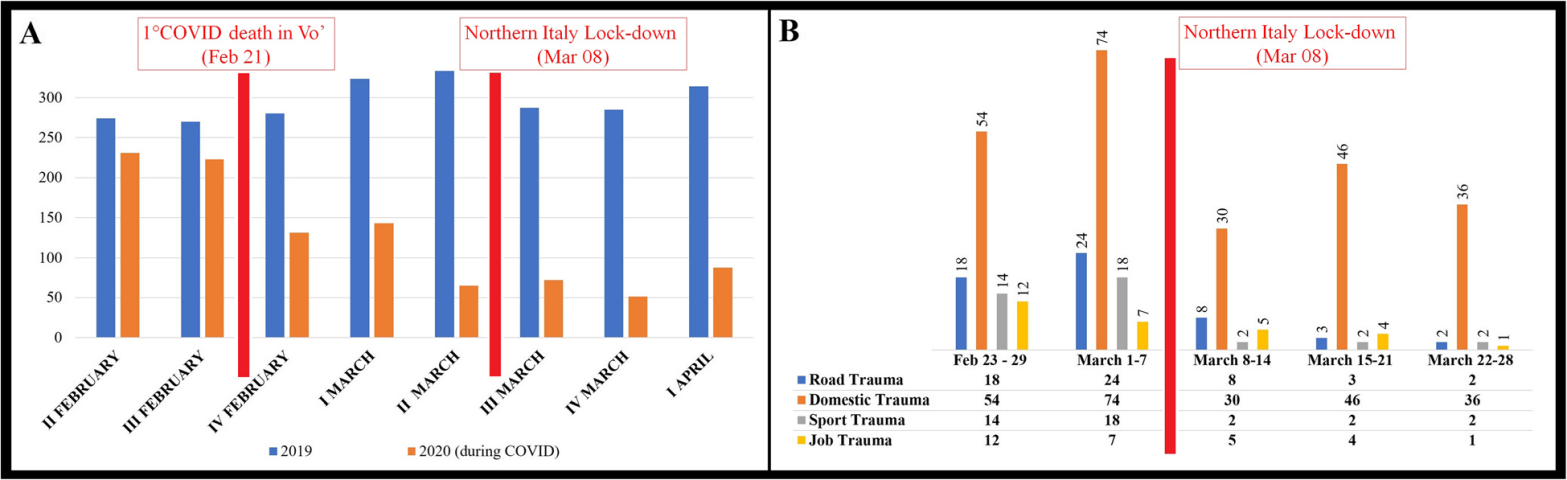
Reduction of orthopedic emergencies. **a** There was a reduction in monthly admission to the orthopedic emergency unit comparing 2019 and 2020. **b** Types of trauma admitted weekly in the orthopedic emergency unit. The practice during COVID-19 in the orthopedic emergency unit showing the remarkable decrease of road, sport, and job traumas

We also had a reduction in outpatient clinic activities (Fig. 2a, b) and admission to the Orthopedic Emergency Unit (Fig. 3a, b).

Philosophy of our current approach on swab testing has proven successful since, of more than 160 people tested in our building, only three COVID-19 positives (all asymptomatic) were found. They were quarantined, and all their potential contacts were identified and periodically checked by swabs (all remained negative). Besides, of over more than 200 surgical procedures, an extremely low incidence of positive patients was found. Only two patients were found COVID-19 positive after discharge (9 and 14 days, respectively). However, their positivity was evident after they were referred to other hospitals for rehabilitation, so possibly they could have been affected by the virus outside our department.

## Discussion

Overall, the evolution and spread of the virus in Italy were dramatically heavy; in fact, the number of diagnosed cases requiring treatment increased rapidly up to today (April 16). Over a population of more than 60,000,000 people, there were 168,941 confirmed COVID-19 positive cases, with 22,170 deaths, 40,164 recovered, and 106,607 with active disease, according to data reported by the Italian Public Safety Committee [1].

**Fig. 4:**
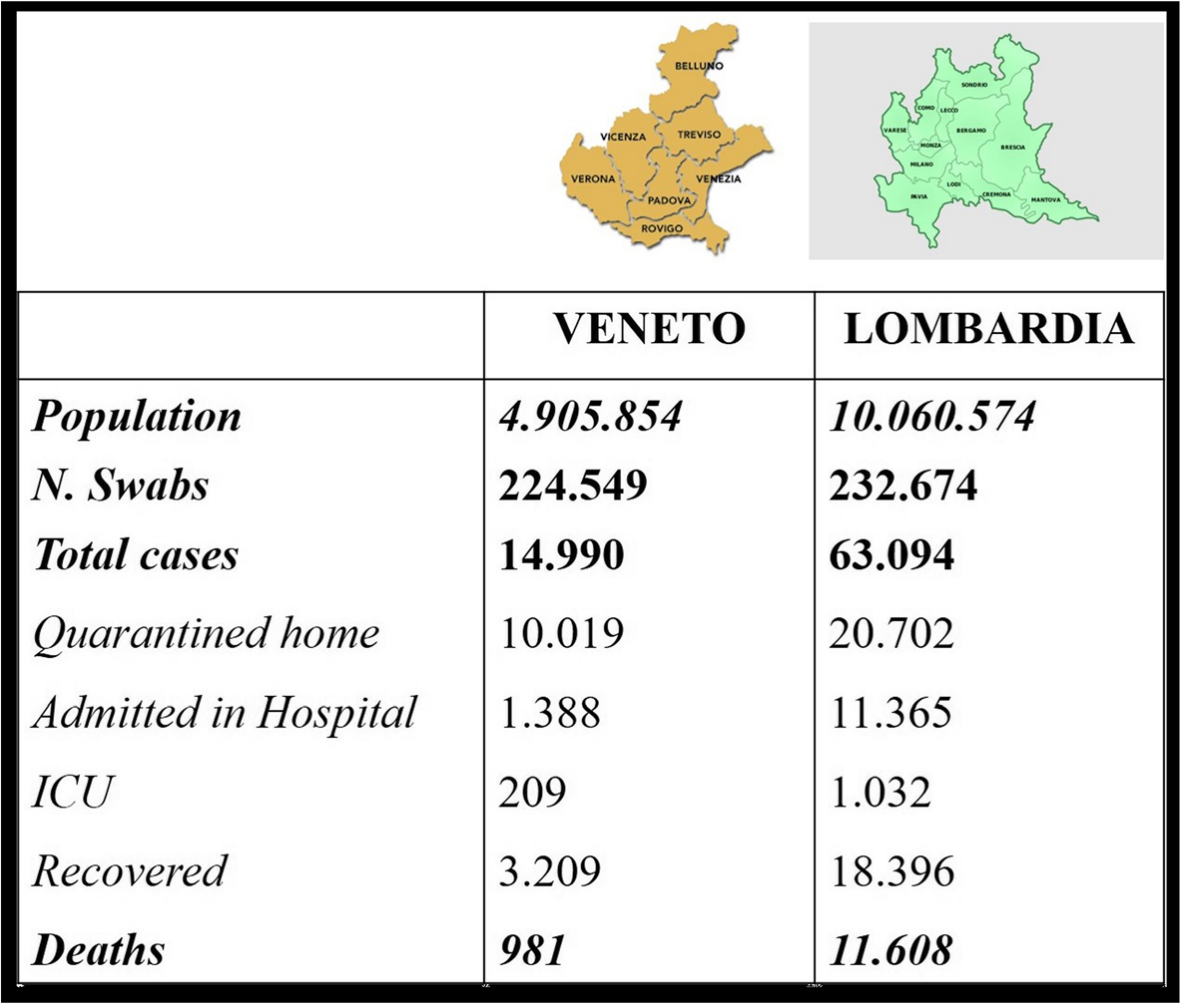
COVID-19 pandemic in Italy comparing virus spread in Veneto and Lombardia

In our opinion, it is particularly essential to retrospectively review the experience in two regions first affected by COVID-19 pandemic: Lombardia and Veneto (Fig. 4). Lombardia accounts for about a double number of population than Veneto; however, there were a quarter of positive cases in Veneto, less than a tenth of deaths, and a similar number than in Lombardia of swab tests performed. Basically, from this data, it is possible to deduce that different strategies were used.

In particular, Veneto strategy was, on one side, an extensive swab test of all people (even if asymptomatic), and on the other, a proactive tracing and quarantining of potential COVID-19 positive patients. On the contrary, in Lombardia, swab tests were performed only in severe symptomatic cases, increasing the risk that asymptomatic patients (possibly COVID-19 positive) could spread the virus in the community. It is particularly true if we consider that all the healthcare staff (physicians, nurses, and sanitary assistants) and all people working in hospitals in Veneto (especially in Padova) were tested and periodically retested with nasopharyngeal swabs. It certainly helped to protect both the healthcare staff and the patients, and it could be the best way up to now, following the experience of South Korea, Singapore, and Hong Kong [2, 3].

**Fig. 5:**
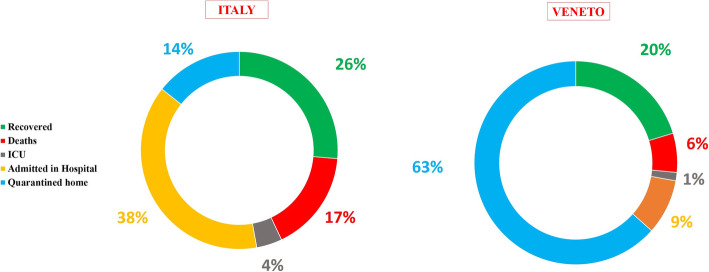
COVID-19 pandemic in Italy comparing Italy overall and Veneto according to 5 different categories of patients (quarantined at home, admitted in hospital, admitted in ICU, dead, and recovered)

Again, when we compare the experience in Italy overall and Veneto, we also may see that the mortality rate in Veneto was lower, as well as the need for hospitalization (Fig. 5). It is probably due to the better denominator of swab testing. Indeed, the swab test’s extensive use gave a more reliable definition of the real situation of the positive patients, leading to a limitation of virus spread from asymptomatic patients [3–7] and the possibility of an earlier start of the treatment. There were fewer cases of COVID-19 patients in the South of Italy: probably it was because chronologically, the first areas affected by COVID 19 were in regions of the North of Italy. So, the Italy lockdown on March 9 was able to prevent virus spread in South Italy; moreover, it could be possible, as suggested by some virologists, that a different climate could play a role in virus spread, although this is not confirmed.

Nevertheless, the overall results in Italy were very poor as they are now in the UK and Spain, and it is known that—even with a very different population—it is becoming delicate, if not dramatic, in the USA.

Our hospital strategy was based firstly on reallocating resources to the treatment of COVID-19 patients and secondly on extensive swab testing. These first action points are similar to those performed worldwide [8]: leading in some hospitals to a redeployment of orthopedics staff (physician and resident), while in others, as well as in ours, to a reduction in orthopedic surgical activities, with the recommendation to perform only acute cases (urgent or emergent), postponing or avoiding elective procedures [2, 9–12]. Changes performed by our medical direction lead to a reduction in our surgical activities. This was also related to a decrease in admission to the Orthopedic Emergency Unit; it was initially determined by the fact that patients voluntarily no longer went to the hospital after the first death in Vo’ Euganeo for fear of contagion (to avoid the risk of virus exposition). Then, the Italian government limited activities and mobility of the people, prohibiting people from having their normal social life and leaving home (Italy lockdown on March 9), with a remarkable reduction of car accidents, bike accidents, etc.

The second action point of our strategy appears successful in minimizing spread to healthcare staff and patients treated in our Orthopedic Department during this period. The key points were the following: First, we treat every suspected case as a potential COVID-19 positive from the start. One OR has been chosen with a specific path and procedure for COVID-19 patients, similar to those reported by other Orthopedic Departments [9, 13]. On the contrary, in other Italian hospitals, swab tests were done only late, showing an incidence of positivity of people working in the orthopedic ORs close or superior to 50% (personal communication, unpublished), similar to what appended in Wuhan at the beginning of the pandemic, where incidence ranged from 1.5 to 20.7% [3].

What does this mean to us? What is the important message that could come to all orthopedic community from our experience? Probably the key to minimizing the extent of the disease and the number of deaths now (in the absence of pathognomonic signs [14], appropriate treatment [15], or of a vaccine) is highlighting all positive patients, social distancing, and quarantining positive patients.

We feel that, if the COVID-19 pandemic persists, in every single hospital, it could be possible to continue the orthopedic surgical activity and also to restart elective surgery [16], using a strategy that implies testing all the healthcare staff and all the patients (possibly before the admission or surgical treatment). This can remarkably contribute to control or minimize the risk of infection spread in the hospital.

## Conclusions

The COVID-19 pandemic is undoubtedly a dramatic situation spreading worldwide, and it is not only affecting people who get infected but also have grave psychological as well as economic consequences in many countries. The future strategy to control the disease or minimize the damages will involve adopting systems to identify possible asymptomatic positive patients and maybe also use apps and digital tools to help recognize positive individuals. Extensive swab test of all people (even if asymptomatic), and a proactive tracing and quarantining of potential COVID-19 positive patients may diminish the virus spread.

The now starting antibodies testing may contribute (doubts still exist about their validity!), and also, at present, it is not known if patients who successfully went through and were cured by infection may even again be re-infected.

It is undoubtedly a significant “war” probably for the healthcare world, and we would like to conclude quoting a sentence from Sir Winston Churchill, “Success is not final, failure is not fatal: it is the courage to continue that counts….”

## Data Availability

Not applicable

## Abbreviations

ICU: Intensive care unit
OR: Operating room
